# Occurrence, molecular characterization, and antimicrobial susceptibility of sorbitol non-fermenting *Escherichia coli* in lake water, fish and humans in central Oromia, Ethiopia

**DOI:** 10.1038/s41598-024-61810-z

**Published:** 2024-05-30

**Authors:** Tesfaye D. Bedane, Bekele Megersa, Fufa Abunna, Hika Waktole, Fanos Tadesse Woldemariyam, Muluken Tekle, Ephrem Shimelis, Fanta D. Gutema

**Affiliations:** 1 Department of Microbiology, Immunology and Veterinary Public Health, Addis Ababa University, P.O. Box 34, Bishoftu, Oromia Ethiopia; 2 Department of Veterinary Science, Salale University, P.O. Box 245, Fiche, Oromia Ethiopia; 3 Armauer Hansen Research Institute, Addis Ababa, Ethiopia; 4 Department of Occupational and Environmental Health, University of Iowa, Iowa City, IA 52246 USA

**Keywords:** Contaminated fish, Lake water, Diarrheic human patients, Sorbitol non-fermenting *E. coli*, Central Ethiopia, Diseases, Health care, Medical research

## Abstract

Contaminated lake water and fish can be sources of bacterial pathogens of public health concern, including pathogenic *E. coli*. Within Ethiopia, specifically, Central Oromia, raw fish consumption is a common practice. Although there are few reports on occurrence of *E. coli* O157 in fish destined for human consumption and children under five years, information on the transmission pathways of *E. coli* O157 and other sorbitol non-fermenting (SN-F) *E. coli* from water-to-fish-to-human, and their virulence factors and antimicrobial resistant determinants along the fish supply chain is lacking. The study aimed to investigate the occurrence, molecular characteristics, and antimicrobial susceptibility of *E. coli* O157 and other SN-F *E. coli* strains in fish, lake water and humans in central Oromia, Ethiopia. A total of 750 samples (450 fish samples, 150 water samples, 150 human stool samples) were collected from five lakes and three health facilities. The samples were processed following the standard protocol recommended by European Food Safety Authority and Kirby–Bauer disc diffusion method for detection of the bacteria, and antimicrobial susceptibility tests, respectively. Molecular characterization of presumptive isolates was performed using Whole-Genome Sequencing** (**WGS) for serotyping, determination of virulence factors, antimicrobial resistance traits, and genetic linkage of the isolates. Overall, 3.9% (29/750) of the samples had SN-F *E. coli;* of which 6.7% (n = 10), 1.8% (n = 8) and 7.3% (n = 11) were retrieved from water, fish, and diarrheic human patients, respectively. The WGS confirmed that all the isolates were SN-F non-O157: H7 *E. coli* strains. We reported two new *E. coli* strains with unknown O-antigen from fish and human samples. All the strains have multiple virulence factors and one or more genes encoding for them. Genetic relatedness was observed among strains from the same sources (water, fish, and humans). Most isolates were resistant to ampicillin (100%), tetracycline (100%), cefotaxime (100%), ceftazidime (100%), meropenem (100%), nalidixic acid (93.1%) and sulfamethoxazole/trimethoprim (79.3%). Majority of the strains were resistant to chloramphenicol (58.6%) and ciprofloxacin (48.3%), while small fraction showed resistance to azithromycin (3.45%). Isolates had an overall MDR profile of 87.5%. Majority, (62.1%; n = 18) of the strains had acquired MDR traits. Genes encoding for mutational resistance and Extended-spectrum beta-lactamases (ESBL) were also detected. In conclusion, our study revealed the occurrence of virulent and MDR SN-F *E. coli* strains in water, fish, and humans. Although no genetic relatedness was observed among strains from various sources, the genomic clustering among strains from the same sources strongly suggests the potential risk of transmission along the supply chain at the human–fish-environment interface if strict hygienic fish production is not in place. Further robust genetic study of the new strains with unknown O-antigens, and the epidemiology of SN-F *E. coli* is required to elucidate the molecular profile and public health implications of the pathogens.

## Introduction

Foodborne diarrheal diseases, including those acquired through fish consumption, are among the leading cause of morbidity and mortality globally with a mortality rate of 22·4 deaths per 100, 000 person-years, and a substantial impairment to socioeconomic development worldwide^1,2^. The mortality rate is higher among children under 5 years, elderly people over 70 years, and in low-income countries^3^.

Perishable food items like ground beef, raw milk, meat, fish, vegetables, unpasteurized fruit juices originated from contaminated sources or contaminated during production or processing may harbor potential foodborne pathogens, including *E. coli*^4–6^. For instance, cross-contamination, inadequate cooking and storage are the dominant food safety impediments responsible for 60–78% of the foodborne disease burden (FBD); while raw foods accounted for an estimated 23–41% of the disease burden in France^7^.

Both wild and cultured fish are sources of a wide variety of bacterial pathogens of public health concern^8,9^. Human infections due to potential pathogens acquired from fish or the aquatic environment are also quite common depending on season, poor hygienic fish handling practices, raw fish consumption habits and the immune status of the exposed individuals^10^; implying that detection of the microbial quality of fish intended for human consumption is crucial^11^.

Although most strains of *E. coli* are normal inhabitants of the intestinal tract of humans, animals and fish^12^, some strains have acquired genetic determinants encoding for various virulence factors giving them the capacity to exert intestinal and extra-intestinal illness in humans with a characteristic watery or bloody diarrhea^13^. Based on their clinical, epidemiological, and pathogenic characteristics, there are seven pathotypes of diarrheagenic *E. coli* strains; including enteropathogenic *E. coli* (EPEC), enterotoxigenic *E. coli* (ETEC), enteroinvasive *E. coli* (EIEC), enterohemorrhagic *E. coli* (EHEC) or Shiga toxin producing *E. coli* (STEC), enteroaggregative *E. coli* (EAEC), diffusely adherent *E. coli* (DAEC)^13^, and a new pathotype, adherent invasive *E. coli* (AIEC)^14^. Each pathotype has a distinctive virulence factor responsible for colonization and subsequent pathogenic effects of the pathogen^15,16^, among which EPEC, ETEC, and EAEC are the dominant causes of infantile diarrhea in developing countries with a relatively low standard of living^17^.

*E. coli* may contaminate fish products destined for human consumption and become a potential fish safety concern^12^. For instance, from 2009–2015 in the United States and Puerto Rico alone, the FBD Outbreak Surveillance System (FDOSS) section of CDC received reports of 344 outbreaks and 2,288 illnesses associated with consumption of contaminated food of aquatic origin including fish^18^. A report from the Zhejiang Province of China has also shown, from 2010–2020 aquatic products including fish were responsible for 109 outbreaks, 1073 cases, and 77 hospitalizations, though no death was recorded^19^. Previous reports have also shown that zoonotic pathogens including *E. coli* can be transmitted to humans via consumption of contaminated and improperly processed fish or fish products^20^.

However, despite increasing evidence of detecting *E. coli* strains harboring many virulence genes from human and animal feces, only a few studies have investigated the occurrence of pathogenic *E. coli* strains in environmental water including lake water^21^, and fish^12^. Although Ethiopia has a huge potential of fish production and its consumption is common in the country, the fish quality and safety aspect is overlooked by regulatory bodies due to limited implementation of food safety regulations^22^. The report of Bedane et al. 2022 indicated lack of infrastructure for fish production and processing, unavailability of cold chain facility for transportation, unhygienic handling practices, and the habit of consuming raw fish in central Oromia, Ethiopia^23^. Besides, some studies reported the occurrence of Shiga toxin producing *E. coli* O157 in fish and humans from various parts of the country. A 1.6% and 1.3% occurrence of *E. coli* O157 in fish in Lake Hayq and Tekeze dam, respectively, was reported from northern Ethiopia^24^. Similarly, Tilahun and Engdawork^25^, have reported 2.3% prevalence of the pathogen from fish in Lake Hawassa, southern Ethiopia. In humans, 15.3% prevalence and 28.9% isolation rate of *E*. *coli* O157 related diarrhea in children under five years was reported from Eastern Ethiopia^26^, and Bahir Dar town of northern Ethiopia^27^, respectively. In a similar study, Gutema et al*.*^28^, have detected *E*. *coli* O157 in 2.8% of 216 diarrheic patients' stool samples investigated in Bishoftu town, central Ethiopia. Besides*, E. coli* is the dominant donor and recipient of resistance genes through horizontal gene transfer, and directly or indirectly, it is the prime cause of treatment failures in both animals and humans. Thus, at a global scale, antimicrobial resistant *E. coli* is a public health concern^29^.

In Ethiopia, Central Oromia in particular, although raw fish consumption is a common practice, and there are few reports of *E. coli* O157 from fish destined for human consumption and children under five years, information on the transmission pathways of *E. coli* O157 and other sorbitol non-fermenting *E. coli* strains along the fish supply chain and, the virulence factors and antimicrobial resistant determinants of the pathogens is lacking. Therefore, this study was aimed to investigate the occurrence, molecular characteristics, and antimicrobial susceptibility of *E. coli* O157: H7 and other sorbitol non-fermenting *E. coli* strains in lake water, fish, and humans to examine potential transmission pathways along fish supply chain in central Oromia, Ethiopia.

## Materials and methods

### Study sites and settings

The study was conducted from December 2020 to June 2022 in East Shewa zone **(**Bishoftu, Koka and Batu**)**. East Shewa zone is located in the upper rift-valley region and endowed with, a number of crater lakes. The zone has a total population of 1,356,342 within an area of 8,370.90 square kilometers with a population density of 162.03. Three-fourths (74.9%) of the population are mixed crop-livestock farmers dwelling in rural settings, while 25.1% are urban inhabitants^30^. The rift-valley lakes including lakes Dambel, Koka, Babogaya, Hora-Arsedi and Koftu are important source of fish for consumers in the adjacent towns (Bishoftu, Koka and Batu towns) and beyond^31^.

### Ethics statement

The study was reviewed and approved by Addis Ababa University Ethical Review Committee (Ref No.VM/ERC/14/05/13/2021) and Oromia Health Bureau Health Research Ethical Review Committee (Ref No. BEFO/4BTW/1-16/10393); and all methods were performed in accordance with ARRIVE guidelines (https://arriveguidelines.org). Moreover, after explaining the purpose of the study, informed consent was obtained from all subjects and/or their legal guardians.

### Study design and sampling

**Figure 1:**
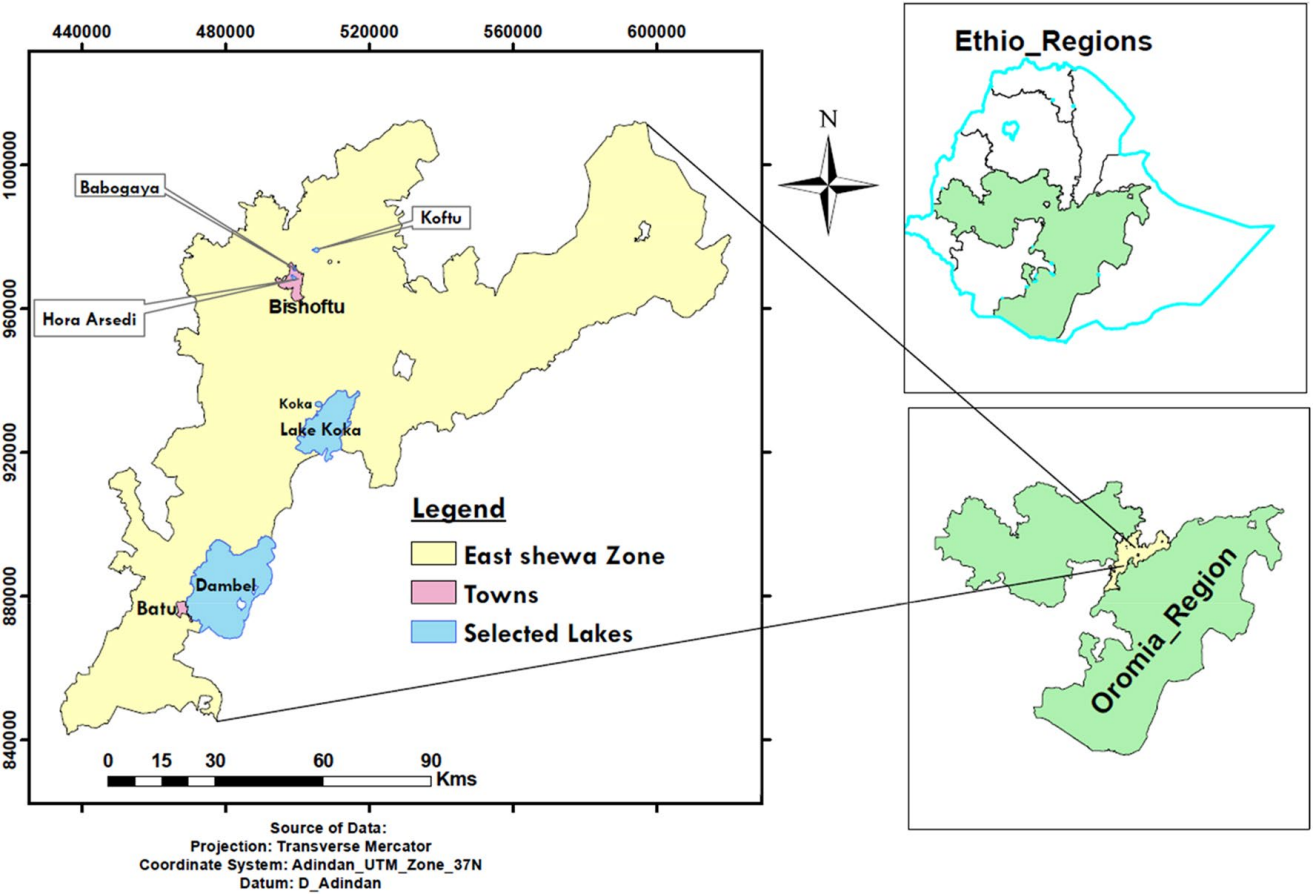
Map of the study areas and the Oromia reginal state within the federal democratic republic of Ethiopia.

A cross-sectional study design was employed to collect data from five lakes that were conveniently selected based on their accessibility and fishing potential. These include Babogaya, Hora-Arsedi and Koftu Lakes in Bishoftu town; Koka Lake nearby Koka town, and Dambel Lake at Batu town. Stool samples were collected from diarrheic out-patients at Bishoftu hospital, Koka health center and Batu health center (Fig. 1). On each sampling day, live fish were purchased at the lake shore from the fishermen who harvested them for commercial purposes, and placed in a bucket of water so that they feel as if they are in their natural environment. Then, the fish were stunned by mixing a clove oil (having an anesthetic nature) into the bucket of water in which they were placed few minutes before severing with a sharp knife to reduce pain during slaughter. Thirty fish from each Lake, comprising of six fish species (*Oreochromis niloticus, Clarias gariepinus,* Tilapia Zilli, *Cyprinus carpio, Labeobarbus intermedius, Barbus ethiopicus*), which are commonly used for human consumption were included in the study (n = 150), from which 150 fecal, 150 meat, 150 skin swabs were sampled. A total of 750 samples, comprising of 150 water samples, 450 fish samples, and 150 stool samples were collected and processed. A maximum of ten fish were identified and sampled per sampling day from which 30 samples consisting of each ten fecal, meat and swab samples were collected on a visiting day.

### Sample collection

Skin swabs were collected using 2 × 3 cm sterile cotton tipped swabs soaked in 10 ml of buffered peptone water (BPW) (Oxoid Ltd., Hampshire, England), as described by McEvoy et al*.*^32^. The skin was swabbed several times first horizontally and then, vertically both in the left and right lateral commissars of the fish from the gill area towards the caudo-ventral region covering the entire dissection area of ~ 10 × 10 cm (an area with substantial risk of contact with a fillet during scaling). Then, the swabs were placed in a sterile universal bottle containing 10 ml of BPW by removing extra shafts and the bottles were screw capped.

After dissection, scaling, and gutting, ~ 10 g of meat (fillet) samples were taken from different parts of the fillet nearby the dissection area (the most suspicious part for external and fecal contamination) and pooled as a single sample; placed in a sterile universal bottle containing 10 ml of BPW.

After gutting, ~ 10 g of fecal samples were collected as described by Elder et al.^33^. Briefly, the whole intestinal organs were separated from the remaining parts of the dissected fish and placed in a sterile plastic bag. Then, the intestinal lumen was opened using a sterile surgical blade and the fecal samples were put into a sterile universal bottle containing 10 ml of BPW.

Water samples (~ 20 ml each) were collected by immersing sterile universal bottles containing 10 ml of BPW into a grossly visible dirty area of the water bodies near the Lake shore with an interval of about 2 m between each sampling points. Similarly, about 10 g of stool samples were collected from the stools submitted by the diarrheic patients for laboratory analysis, into a sterile universal bottle containing 10 ml of BPW in collaboration with the laboratory technicians using a sterile toothpick for each sampling. The procedures were done as aseptic as possible using a sterile disposable glove to avoid the risk of cross contamination during each sampling. Finally, all samples were carefully labeled using a permanent marker, kept in an ice box containing ice packs and transported to Addis Ababa University College of Veterinary Medicine and Agriculture Veterinary Public Health Laboratory for processing.

### Detection of sorbitol non-fermenting *E. coli *and* E. coli* O157

The samples were primarily processed targeting sorbitol non-fermenting *E. coli,* and then Shiga toxin producing *E. coli* O157 strain. Briefly, all the samples in the universal bottle containing 10 ml BPW were incubated at 41.5°c for 6 h to revive and increase the recovery rate of stressed bacterial cells^34^. Then, the meat samples were blended using stomacher (Seward Stomacher 400, Seward, London, UK) at a speed of 150 rpm for 60 s and, the aliquots from the meat, water, fecal, stool and swab cultures were selectively enriched by measuring 10 ml of samples into 90 ml modified tryptone soya broth (mTSB) (Oxoid, Hampshire, England) and agitating using the same blending machine at 150 rpm for 60 s. After homogenization, the selectively enriched samples were incubated overnight at 41.5°c.

A loop-full of aliquots from selective enrichment was streaked on cefixime-potassium tellurite Sorbitol MacConkey agar (CT-SMAC) and incubated at 37°c for 24 h. After incubation, sorbitol non-fermenting colonies (colorless colonies) were sub-cultured on CT-SMAC and incubated at 37°c for 24 h to obtain adequate number of pure colonies. To rule-out the growth of other Gram negative organisms with colorless colonies on CT-SMAC, including *Burkholderia*, *Vibrio, Proteus*, *Klebsiella*, *Aeromonads* and *Pseudomonas*^35^, the pure colonies on CT-SMAC were further sub-cultured onto eosin methylene blue (EMB) agar and incubated at 37°c for 24 h. Isolates with typical characteristic of green metallic sheen color of the generic *E. coli* on EMB agar were further tested for indole production. All indole positive presumptive colonies were preserved on tryptone soya agar after incubating at 37°c for 24 h and adding 80% glycerol for further analysis. The presumptive isolates were shipped to Belgian National Reference Centre, Brussels, Belgium for immunological testing and whole genomic sequencing.

### E. coli O157 latex agglutination test

At the reference laboratory in Brussels, Belgium, the isolates were further sub-cultured on CT-SMAC to perform latex agglutination test. Then, each presumptive isolate was emulsified with normal saline on disposable reaction cards provided in the kit and mixed with a drop of test latex (a latex particle sensitized with specific rabbit antibody reactive with the O157 somatic antigen) and agitated for one minute. After one minute, the result was interpreted based on the standard protocol described by DeBoer and Heuvelink^36^. Due to lack of reference strains, known positive controls were not used. However, after the agglutination test, a suspension of inactivated *E coli* O157 and E *coli* O116 cells in buffer were used as a positive and negative controls, respectively.

### Whole-genome sequencing (WGS)

Genomic DNA was extracted from pure cultures of SN-F *E. coli* isolates grown overnight on SMAC Agar; and its purity and quantity were measured with a Qubit double stranded DNA (dsDNA) BR assay kit. Then, fragmentation of 500 ng of genomic DNA was carried out using the NEBNext® Ultra™ II FS module. Sequencing libraries, with an insert size of on average 550 bp, were prepared using a KAPA Hyper Plus kit (Kapa Biosystems, Wilmington, USA) and a Pippin Prep (Sage Science, Beverly, MA, USA) size selection with a CDF1510 1.5% agarose dye-free cassette. Every sample was assigned an in-house truseq style adapter with a unique dual-indexed 8-bp barcode. PCR amplification (6 cycles) of the library was performed using the KAPA HiFi HotStart Libray Amplification kit (Kapa Biosystems, Wilmington, USA) according to the manufacturer’s instructions. After equimolar pooling, libraries were sequenced on a Novaseq 6000 instrument (Illumina, San Diego, CA, USA) using a NovaSeq 6000 SP Reagent kit (500 cycles) generating 2 × 250 bp reads. For this, the library was denaturated and diluted according to the manufacturer’s instructions. A 1% PhiX control library was included in each sequencing run. Lastly, the raw reads were uploaded, and de novo assembled, using SPAdes v.3.15.3, in BioNumerics v.8.1., and sequence quality was assessed using the quality metrics incorporated in BioNumerics v.8.1.

### Comparison of SN-F *E. coli* strains retrieved from water, fish and humans in EnteroBase

The raw reads of 29 SN-F *E. coli* genomes were uploaded and automatically assembled in the public genome database, EnteroBase. All genome assemblies were subsequently compared to the available *E. coli* genomes using hierarchical clustering of cgMLST (HierCC) at different levels of resolution, ranging from HC0 (hierarchical clusters consisting of identical genomes with no AD) to HC200 (hierarchical clusters consisting of genomes with up to 200 ADs)^37^. However, up on quality check, the genomes of six isolates were poor and only 23 isolates were included in the cluster analysis.

### In silico identification of genes encoding for serotype, virulence factors, and antimicrobial resistance traits

The *E. coli* genotyping tool v.2.1, available in BioNumerics v.8.1 was used to predict *E. coli* serotypes, virulence gene profiles, acquired resistance genes, and point mutations starting from the genome assemblies. The presence of virulence and resistance genes was determined with a minimum % identity (ID) threshold of 85% and a minimum length for coverage of 60%.

### Antimicrobial susceptibility test

Antimicrobial susceptibility test was conducted on a total of 29 SN-F *E. coli* strains retrieved in the present study following the standard protocol described by CLISI 2022^38^, using ten essential antimicrobial agents obtained from commercial market (Thermo Scientific Fisher); namely, (ampicillin 10 μg, tetracycline 30 μg, ciprofloxacin 5 μg, azithromycin 15 μg, chloramphenicol 30 μg, ceftazidime 30 μg, cefotaxime 30 μg, meropenem 10 μg, sulfamethoxazole /trimethoprim 25 μg, and nalidixic acid 30 μg) grouped under eight antimicrobial classes using Kirby-Bauer disc diffusion method^38^. The antibiotics were selected based on their common usage in humans and animals and AMR reports. The discs were funded by Michigan State University through the 2019 faculty exchange program for one of our co-authors. To estimate the concentration of the isolates in the culture broth, 0.5 McFarland Standard (≈ 1.5 × 10^8^ CFU/ml) was prepared by measuring 0.05 ml of 1% BaCl_2_ and 9.95 ml of 1% H_2_SO_4_; and the turbidity of the culture broth was adjusted towards this standard using sterile saline solution. A sterile cotton swab was immersed into the broth culture and uniformly swabbed to Mueller–Hinton agar plates. The plates were allowed to dry for few minutes, and the antimicrobial discs were randomly placed on the surface of the agar plates with gentle pressure using sterile forceps. The plates were incubated at 37 °C for 18 h; and after incubation, the inhibition zones of each antimicrobial agent were carefully measured using a digital caliper. The test results were qualitatively interpreted as susceptible, intermediate, or resistant based on zone diameter interpretative standards established for *E. coli* and other enteric Gram negative rods^38^. Controls were accomplished with an *in-silico* identification of genes encoding for antimicrobial resistance traits using *E. coli* genotyping tool v.2.1, available in BioNumerics v.8.1 to predict acquired resistance genes, and point mutations following the standard procedure described by Feldgarden et al.^39^.

### Data analysis

The data were entered into an excel spreadsheet (Microsoft office® excel 2013, Cengage Learning, version 15) and analyzed using Stata version 15.0 software (Stata Corp, College Station, TX). Descriptive statistics such as frequency and percentages were used to express the sociodemographic characteristics of the diarrheic patients and the antimicrobial susceptibility profiles of the isolates*.* The proportions of occurrence of sorbitol non-fermenting *E*. *coli* in diarrheic patients and, water and fish samples were calculated by dividing the number of culture positive samples by the total number of samples tested from each sample source. Fisher’s exact test was used to assess the difference in the proportion of sorbitol non-fermenting *E*. *coli* among the different sample sources.

The assembled sequencing data was analyzed using the *Escherichia* /*Shigella* cgMLST typing scheme in BioNumerics v.8.1 (core Enterobase). Both assembly algorithms, namely, the assembly-free k-mer-based approach using the raw reads and the assembly-based BLAST approach were used for allele calling. The default settings were used for both the assembly-free and assembly-based algorithms. The quality of the assembly-free and the assembly-based allele calls were verified using the quality statistics window in BioNumerics. The MLST profile of each isolate was determined using the three basic allele mapping experiments (Pub (Achtman) MLST, Pasteur MLST, Whittam MLST) incorporated in BioNumerics. Minimum spanning tree (MSTs) diagrams of the cgMLST data were generated using the MSTree V2 algorithm and visualized by GrapeTree in EnteroBase.

## Results

### Demographic characteristics and proportion of SN-F *E. coli* strains per sample sources

Among the total of 150 diarrheic patients participated in the study, 52.67% of them were males. The mean age of the patients was 30.7 years (range: 9 months to 70 years). From the patients investigated, 8.7% (13), 17.3% (26), 17.3% (26), and 56.7% (85) were observed with the clinical history of bloody, mucoid, mixed and watery diarrhea, respectively (Table 1).

**Table 1:** Demographic characteristics and proportions of samples positive for SN-F *E. coli* per sample sources.

Demographic characteristics	Health facilities	Number of participants	Gender		Average age
			Male	Female	
	Bishoftu Hospital	50	31	19	29.2
	Koka Health Center	50	29	21	32.6
	Batu Health Center	50	19	31	30.4

Sample types	No of samples	Positive, n (%)	χ^2^ (*p*-value)		
Water	150	10 (6.7)	14.0 (0.001)		
Fish	450	8 (1.8)			
Human stool	150	11 (7.3)			

The overall culture-based proportion of sorbitol non-fermenting *E. coli* strains in all samples was 3.9% (29/750). Specific sample level detection rate was 6.7% (10/150), 1.8% (8/450), and 7.3% (11/150) in water, fish, and human samples, respectively (Table 1).

Among the total of 10 SN-F *E. coli* strains detected in water samples, higher proportion (40%, n = 4) was detected in samples collected from lake Koftu, followed by lake Hora-Arsedi (30%; n = 3). Similarly, from a total of 450 fish samples examined, only eight SN-F *E. coli* strains were detected (n = 8; 1.8%, 95%CI: 0.7, 3.5%). The majority, 87.5% (n = 7), of the isolates were recovered from fish collected from lake Koka. Fifty percent (n = 4) of the fish isolates were detected in meat samples, as compared to the skin swab and fecal samples. Nevertheless, none of the fish samples collected from Babogaya, Koftu and Dambel Lakes were tested positive (Table 2).

**Table 2:** Proportion of samples positive for sorbitol non-fermenting *E. coli* per sample types.

Sample type	Source lake	Number of samples	Positive n (%)
Water	Hora-Arsedi	30	3 (30)
	Babogaya	30	1 (10)
	Koftu	30	4 (40)
	Koka	30	1 (10)
	Dambel	30	1 (10)
Fish samples	Babogaya	90	0 (0)
	Hora-Arsedi	90	1 (1.1)
	Koftu	90	0 (0)
	Koka	90	7 (7.8)
	Dambel	90	0 (0)

	Age (years)	Number	Positive n (%)
Human stool	Children under 5	12	1 (8.3)
	Young (5–17)	19	2 (10.5)
	Youth (18–30)	49	4 (8.2)
	Adults (≥ 31)	70	4 (5.7)

In diarrheic patients, the overall detection rate of sorbitol non-fermenting *E. coli* strains was 7.3% (95% CI: 3.7, 12.7%) with higher prevalence in younger age groups (5–17 years) and children under 5 years; at a detection rate of 10.5% and 8.3%, respectively (Table 2).

Core genome multi locus sequence typing (cgMLST) confirmed that all the strains detected from water, fish and humans were SN-F *E. coli* strains. None of the *E. coli* strains were tested positive for *E. coli* O157 using *E. coli* O157 latex agglutination test, and whole genome sequencing (WGS). Core genome MLST also detected a new strain with unknown O-antigen from fecal sample of fish obtained from Lake Koka and stool sample of diarrheic patient presented to Bishoftu Hospital (Table 3).

**Table 3:** Antigenic characteristics and MLST of SN-F *E. coli* strains retrieved from water, fish, and humans.

Sample type	Source	Antigenic characteristics	MLST Pasteur ST	MLST Whittam ST	MLST PubMLST (Achtman) ST	Number of Isolates with the Same Antigen
Water	Lake Babogaya	O116: H49	publicST325	ST2001	publicST2520	1
	Lake Hora Arsedi	O20: H8	ST2121	ST611	publicST164	1
	Lake Hora Arsedi	O174: H43	ST2119	ST2001	publicST2244	1
	Lake Koftu	O116: H49	publicST325	ST2001	publicST2520	1
	Lake Koftu	O8: H10	publicST86	ST2608	N/A	1
	Lake Koka	O17/O44: H18	ST2118	ST2607	publicST69	1
	Lake Dambel	O17/O44: H18	ST2118	ST2607	publicST69	1
Fish skin swab	Lake Koftu	O176: H11	ST2120	ST1634	publicST48	2
Fish Meat	Lake Koka	O176: H11	ST2120	ST1634	publicST48	1
	Lake Koka	O155: H21	publicST87	ST301	publicST58	1
	Lake Koka	O10: H5	ST785	ST2605	publicST206	1
	Lake Babogaya	O18ac: H21	ST2123	ST2611	publicST223	1
Fish Feces	Lake Koka	O-Unknown: H28	ST2122	ST2609	publicST1633	1
Human stool	Koka Health Center	O168: H2	ST2117	ST2606	N/A	2
	Koka Health Center	O8: H25	publicST24	ST294	publicST58	1
	Bishoftu Hospital	O-Unknown: H40	publicST2	ST2610	publicST10	1
	Bishoftu Hospital	O155: H10	publicST21	ST284	publicST1049	1
	Batu Health Center	O155: H10	publicST21	ST284	publicST1049	4

### Comparison of SN-F *E. coli* Genomes in EnteroBase

**Figure 2:**
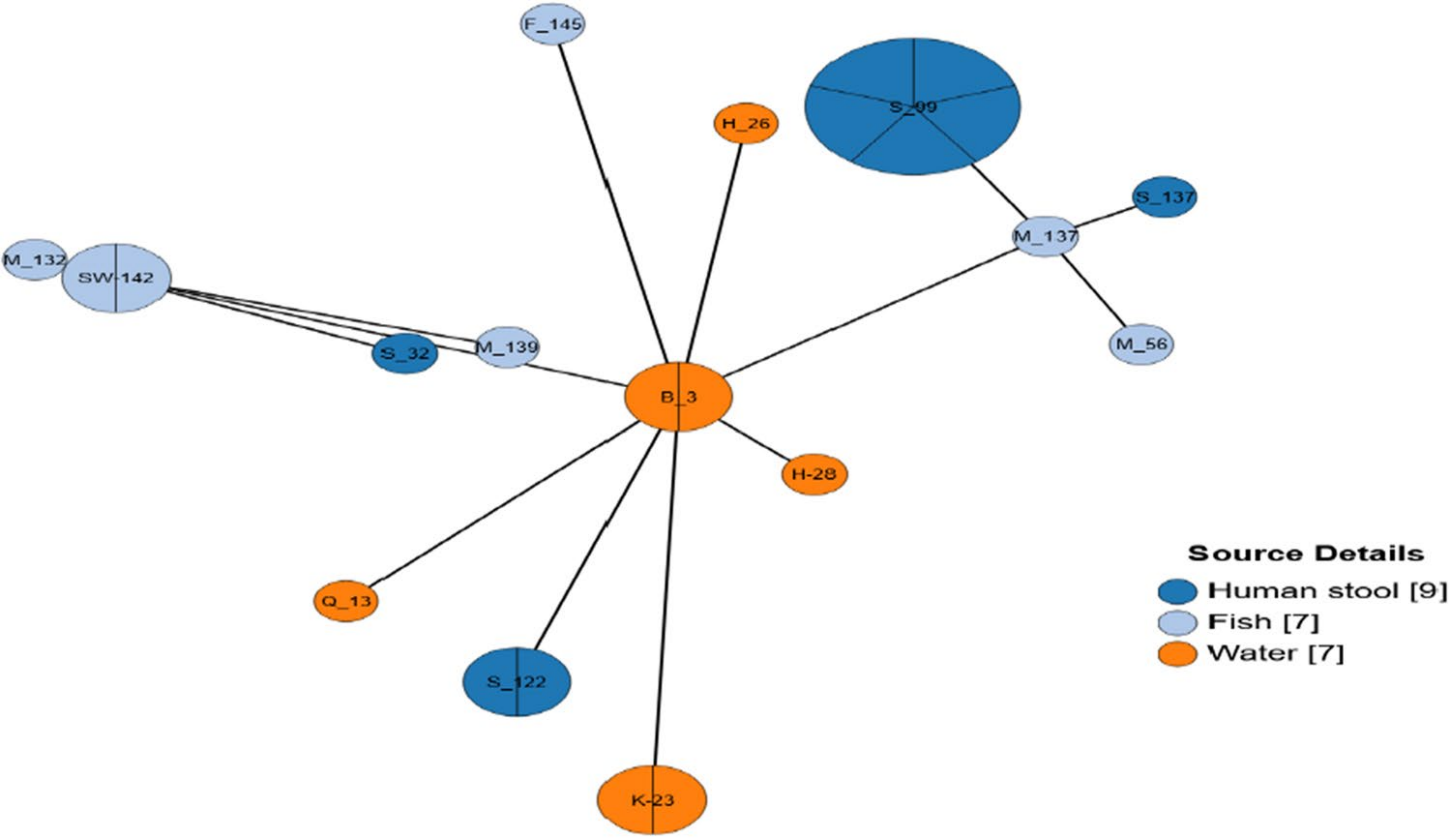
Grape-Tree diagram generated in EnteroBase based on core genomic MLST profiles of 23 *E. coli* strains isolated from fish, lake water and humans showing lack of clustering among strains from the three sample sources. Nodes are color-coded based on the sample types and sample ID.

Comparison of the genetic linkage among the SN-F *E. coli* strains retrieved from the three sample sources (water, fish and humans) in EnteroBase showed lack of genetic relationships among the isolates. However, genetic relatedness was observed among strains from the same sample sources: 2 clusters of *E. coli* strains from humans (5 and 2 strains), water (2 strains) and Fish (2 strains) (Fig. 2).

### In silico identification of genes encoding for virulence factors and antimicrobial resistance traits

An in silico MLST showed that all the strains have multiple virulence factors and one or more genes encoding for them. The most repetitive virulence factors and respective encoding genes detected were survival (*iss*), adherence proteins (*papC, lpfA, irp2, k88ab*), invasion (*ompT, cia*), toxin production (*hlyE, hlyF, cvaC, mchF*), and metabolic regulation (*terC, eilA*). It also revealed genes encoding for acquired resistance of ampicillin, amoxicillin, cephalothin, piperacillin, and ticarcillin (*blaTEM-1B*), doxycycline and tetracycline (*tet(A)*), trimethoprim (*dfrA1*and *dfrA5*), sulfamethoxazole (*sul1*), fosfomycin (*fos7*), chloramphenicol (*catA1*), ciprofloxacin (*qnrS*1), and nalidixic acid (*gyrA*). Moreover, genes encoding for mutational resistance against ciprofloxacin and nalidixic acid ((*parC, gyrA*), and extended spectrum beta-lactamase (ESBL) against aztreonam, cefepime, ceftaxime, ceftazidime, and ceftriaxone (*blaCTX-M15*) were detected (See the supplementary file).

Overall, 18 (62.1%) of the isolates were virulent strains; of which, 7 (38.9%), 6 (33.3%), and 5 ((27.8%) were retrieved from water, fish, and human, respectively. The majority (55.6%; n = 10) of the virulent isolates have genes encoding for antimicrobial resistance. Among this, 5 (50%), 4 (40%) and 1 (10%) isolates were from humans, fish and water respectively (See the supplementary file).

### Antimicrobial susceptibility

Of the 29 sorbitol non-fermenting *E. coli* strains tested, 96.6% (n = 28) of them were susceptible to azithromycin followed by ciprofloxacin (20.7%, n = 6), chloramphenicol (17.3%, n = 5) and sulfamethoxazole /trimethoprim (13.8%, n = 4). All of the isolates were resistant to ampicillin, cefotaxime, ceftazidime, meropenem and tetracycline (Table 4).

**Table 4:** Antimicrobial susceptibility profiles of sorbitol non-fermenting *E. coli* strains.

Antimicrobial classes	Antimicrobial agents	Concentration (μg)	Susceptibility		
			Susceptible n (%)	Intermediate n (%)	Resistant n (%)
Penicillins	ampicillin	10	0 (0.0)	0 (0.0)	29 (100)
Macrolides	Azithromycin	15	28 (96.6)	0 (0.0)	1 (3.45)
Cephalosporin	Cefotaxime	30	0 (0.0)	0 (0.0)	29 (100)
	Ceftazidime	30	0 (0.0)	0 (0.0)	29 (100)
Chloramphenicol	Chloramphenicol	30	5 (17.3)	7 (24.1)	17 (58.6)
Quinolones	Ciprofloxacin	5	6 (20.7)	9 (31)	14 (48.3)
	Nalidixic acid	30	0 (0.0)	2 (6.9)	27 (93.1)
Carbapenems	Meropenem	10	0 (0.0)	0 (0.0)	29 (100)
Tetracyclines	Tetracycline	30	0 (0.0)	0 (0.0)	29 (100)
Sulphonamides	Sulfamethoxazole/trimethoprim	25	4 (13.8)	2 (6.9)	23 (79.3)

A multi-drug resistance (MDR) of 87.5% was observed; where the strains were resistant to seven antimicrobial classes from the eight antimicrobial classes tested. Five isolates and one isolate were resistant to 90% (n = 9) and 100% (n = 10) of the antimicrobial agents used, respectively. An in silico MLST analysis also confirmed that the majority (62.1%; 18/29) of the strains have multiple acquired resistance against ampicillin, amoxicillin, cephalothin, piperacillin, ticarcillin, doxycycline, tetracycline, trimethoprim, sulfamethoxazole, and one or more genes encoding for them. Genes encoding for mutational resistance against ciprofloxacin and nalidixic acid, and extended spectrum beta-lactamase (ESBL) against aztreonam, cefepime, ceftaxime, ceftazidime, and ceftriaxone were also detected (See the supplementary file).

## Discussion

In this study, we investigated the occurrence, molecular characteristics, and antimicrobial susceptibility of sorbitol non-fermenting *E. coli* with particular emphasis to *E. coli* O157 in Lake Water, fish and humans in central Oromia, Ethiopia. The study showed that none of the identified isolates are *E. coli* O157. Interestingly, the study revealed antimicrobial resistant SN-F *E. coli* and new SN-F *E. coli* strains with unknown O-antigen, and genetic relatedness among strains from the same sources with no linkage among strains from different sample sources (water, fish and humans). The majority of the strains have multiple genes encoding for a single virulence factor and antimicrobial resistance traits; implying that the isolates are pathogenic and antimicrobial resistant *E. coli* strains.

The genetic relationships among the isolates from the same sources suggests the circulation and the potential risk of dissemination of virulent and antimicrobial resistant strains along the fish supply chain in the study area. Similar report from Southeastern Nigeria revealed the occurrence of MDR SN-F *E. coli* strains in other species of animals (broiler chicken, cattle and pig), and suggested that the pathogens may spread from animals to humans and the environment making it a public health threat^40^. Likewise, the recent report of Bedane et al.^23^, on hygienic fish handling practices in the study area indicated that the Lakes are used as watering points for cattle, which are the principal carriers of *E. coli* O157:H7, and fully accessible to run-off water, which may carry many other pathogens of public health concern, including SN-F *E. coli*.

Reports on SN-F non- O157 *E. coli* strains are limited and as a result, our findings are discussed in relation to previous reports on other *E. coli* strains and *E. coli* O157 as appropriate to provide an overall insight. The observed 6.7% SN-F *E. coli* strains in lake water samples in the present study is lower than the finding of Mekonnen et al.^41^, who reported 93.3% *E. coli* in water samples collected from Lake Dambel, and nearly comparable with the report of Dissasa et al.^42^, who retrieved 5.9% of generic *E. coli* from water samples of Lakes Dambel, Langano and Hawasa of Ethiopia. Similarly, Akoachere et al.^43^, in Cameron and Ribeiro et al.^44^, in Brazil found that bacteria of the family Enterobacteriaceae, including *E. coli*, are widely distributed in the aquatic ecosystem. Once the aquatic environment is contaminated with bacterial pathogens of public health concern, fish can easily acquire the pathogens naturally by direct physical contact^44^. This substantiates the higher odds of exposure of fish to pathogenic organisms and the subsequent higher odds of human exposure to the pathogens. The observed variations could be due to differences in the method of detection and targeted *E. coli* strains in this study. We primarily investigated the occurrence of sorbitol-non fermenting *E*. coli as compared to other studies that reported the prevalence of the generic *E. coli* which yield higher detection rate. Moreover, the variations could be attributed to the temporo-spatial disparity of the studies, and related anthropogenic and natural elements which affect the magnitude of contamination of lake water.

Sorbitol non-fermenting *E. coli* strains were detected in 1.8% of fish samples analyzed during the present study. An important notable finding is that 50% of the fish isolates were detected in meat, showing a remarkable contamination of the filet either from the external environment including skin, or intestinal contents due to poor handling and processing practices. Similar study conducted in India reported 19.2% occurrence of SN-F *E. coli* in cattle feces^45^, implicating the potential risk of contamination of Lake water and fish with SN-F *E. coli* strains of cattle fecal origin. The poor hygienic conditions along the fish supply chain in the study area might have contributed to the contamination of the lakes with animal feces during watering and through water run-off^23^.

Compared to the previous studies by Haile and Getahun^46^, Yohans et al.^47^, and Dissasa et al.^41^, who reported the generic *E. coli* in 12%, 20%, and 5.7% of fish samples collected from Lakes Dambel, Tana, and three rift-valley Lakes (Dambel, Langano and Hawasa), respectively, our report is exceedingly lower. It is also extremely lower than the finding of Marijani^48^, who recovered *E. coli* from 39% of fish sampled from marine and freshwater fish in Tanzania. Our detection of sorbitol non-fermenting *E. coli* on the edible part of fish agrees with the findings of Akoachere et al.^43^, from Cameron, and Ribeiro et al.^44^, from Brazil who previously recovered *E. coli* from fish destined for human consumption. The report of Anyanwu et al.^40^, from Nigeria have also shown 13.4% prevalence of SN-F *E. coli* in other food animals (broiler chicken, cattle and pigs), among which 0.51% of them were *E. coli* O157.

The 7.3% occurrence of SN-F *E. coli* strains in diarrheic patients is lower than the systematic review and meta-analysis findings of Zenebe et al*.*^49^, who reported 25% pooled prevalence of a generic *E. coli* in diarrheic children under five years in Ethiopia, and 15.3% prevalence and 28.9% isolation rate of *E*. *coli* O157 related diarrhea in children under five years from Eastern Ethiopia^26^, and Bahir Dar town of northern Ethiopia^27^, respectively.

The occurrence of highly virulent MDR *E. coli* strains in humans may be related to unhygienic handling of animal products including fish or consumption of raw or undercooked products. Likewise, the reports of Haenen et al.^50^, Santos and Vieira^51^, and Mekonnen et al.^52^, showed unhygienic handling and consumption of raw or undercooked infected fish may pose the risk of infection in susceptible individuals. Similarly, Wiriyaprom et al.^53^, have reported 8.82% prevalence of SN-F Shiga toxin-producing *E. coli* (STEC) isolated from goats in Thailand; among which only 0.77% was *E*. *coli* O157.

The majority, (62.1%) of the strains have virulence determinants encoding for adhesion, host invasion, toxin production, and promoting survival of the pathogen. In addition, genes encoding for deterioration of metabolic regulation and iron uptake of the susceptible hosts were detected in majority of the isolates retrieved from the three sample sources, implying that the strains are sharing many virulence characteristics. A similar study in Mozambique also showed the coexistence of different combinations of two or more virulence genes encoding for a single virulence factor in *E. coli* strains retrieved from ready to eat food items^54^.

Sorbitol non-fermenting *E. coli* strains carrying virulence determinant traits were detected in all the study lakes, all health facilities and fish sampled from lakes Koka and Koftu. Among them, a higher proportion, (38.9%; n = 7) was detected from water samples; suggesting that the pathogen is exceedingly disseminated across the aquatic ecosystem in the study areas.

The higher prevalence of virulent strains in lake water implies that fish can be easily infected with such virulent strains from the aquatic environment, and the pathogens can be disseminated among the fish population in that ecosystem and become a potential public health threat, especially among raw fish consumers. Previous reports also showed that if the water bodies are contaminated with pathogenic organisms, fish can be easily infected from its immediate environment^55^.

The new SN-F *E. coli* strains with unknown O-antigens, O-unknown: H28 and O-unknown: H40, from fish and human, respectively might be mutant progenies of known *E. coli* strain or newly emerging strains not yet characterized. Moreover, in silico MLST have shown that both the new strains with unknown O-antigen have multiple virulence factors, and O-unknown: H40 is MDR strain. Thus, further robust molecular study is required to determine the lineages of these strains relative to *E. coli* strains in the sequence database to elucidate fundamental information on the epidemiology and public health implications of the strains. Similar previous studies conducted on bacterial genomics have also shown that comparison of bacterial 16S rRNA gene (the most conserved genetic marker of a bacteria) with a known sequence of related bacteria in the database has emerged as a preferred genetic technique to identify new strains^56,57^.

Among the ten antimicrobial agents tested for their level of efficacy, azithromycin was effective for 96.6% (n = 28) of the isolates and recognized as the most promising drug for the treatment of infections related to SN-F non-O157 *E. coli* strains. Studies indicated that azithromycin is the most promising alternative and excellent drug for the treatment of diarrhoeagenic enteric infections caused by *E. coli*, Shigella, Salmonella, and Campylobacter species^58–60^. Reduced efficacy of ciprofloxacin, chloramphenicol and sulfamethoxazole /trimethoprim to SN-F non-O157 *E. coli* strains was observed as compared to the efficacy of azithromycin.

The lower efficacy of ciprofloxacin and sulfamethoxazole /trimethoprim is in agreement with the current report of Yasmin et al.^61^, from Pakistan, who reported 93% and 92% resistance of *E. coli* strains to the two drugs, respectively. Other study reported a better and moderate efficacy of ciprofloxacin and sulfonamides to *E. coli* strains recovered from neonates in China^62^. On the other hand, the low efficacy of chloramphenicol to SN-F non-O157 *E. coli* strains is lower than the finding of Ashenafi et al.^63^, who reported 27.3% efficacy of the drug to *E. coli* O157 retrieved from raw cow milk in central Ethiopia.

Conversely, in the present study, it was noted that all isolates are resistant to five antimicrobial agents, namely: ampicillin, cefotaxime, ceftazidime, meropenem and tetracycline; and besides all human isolates were resistant to nalidixic acid. These findings are comparable with the recent findings of Dejene et al.^64^, from Ethiopia, who have reported an absolute resistance of *E. coli* O157 to ampicillin, and Yasmin et al.^61^, from Pakistan, who have shown 91% resistance of other *E. coli* strains to the same drug. Thus, based on the result of the present study and previous findings, avoiding the use of ampicillin for the treatment of *E. coli* O157 and other *E. coli* infections is recommended. The absolute resistance to cefotaxime, ceftazidime and meropenem observed in the present study is also comparable with the finding of Yasmin et al.^61^, who reported a 98%, 86% and 71% resistance of *E. coli* strains to the same drugs, respectively.

The study revealed the widespread occurrence of MDR SN-F *E. coli* strains along the fish supply chain in central Oromia, Ethiopia, where the strains were resistant to 87.5% (seven-out of eight) antimicrobial classes. Based on in silico MLST, the majority (55.6%) of the virulent strains, have multiple acquired antimicrobial resistant traits and one or more genes encoding for them. This implies the higher AMR profile of virulent strains retrieved from humans as compared to fish and water isolates. Similar report from the city of Maputo, Mozambique have also shown that MDR *E. coli* strains was detected in drinking water^54^. Another study reported MDR Gram-negative rods, including *E. coli* as a public health threat globally^65^. Thus, as MDR Gram-negative bacteria, including *E. coli*, represent a global public health challenge^66^, it is crucial to note that these resistant organisms may be disseminated along the fish supply chain, and become a potential public health threat. Moreover, genes encoding for mutational resistance against ciprofloxacin and nalidixic acid, and extended spectrum beta-lactamase (ESBL) against aztreonam, cefepime, ceftaxime, ceftazidime, and ceftriaxone were detected. Besides, previous reports have shown that *E. coli* strains may serve as reservoir of antimicrobial resistant genes which can be transferred to other pathogenic strains primarily through horizontal gene transfer^29^, and become a serious public health concern^67^. Therefore, since they are highly prone to exchanging genetic material^68^, the risk of horizontal gene transfer to other pathogenic or non-pathogenic *E. coli* strains and dissemination of mutant strains along the fish supply chain may be inevitable.

In general, although resistance of *E. coli* to older antibiotics such as tetracycline and ampicillin and those that acquire resistance through plasmid transfers may not be surprising, resistance to the last-resort drugs like meropenem which are used for the treatment of human infections caused by MDR enteric pathogens and classified by WHO as high priority critically important antimicrobials^69^, is a potentially discerning public health threat; and rational use of drugs are critically important.

## Conclusion

The study revealed the occurrence of considerable proportion of virulent and multidrug resistant sorbitol non-fermenting *E. coli* strains in Lake Water, fish and humans in Central Oromia, Ethiopia. We also reported new SN-F *E. coli* strains with unknown O-antigen from fish and stool samples. However, the strains detected from the three sample sources are not genetically linked; and none of them were *E. coli* O157. The observed occurrence of virulent and multidrug resistant *E. coli* strains and the genetic linkage among strains originated from the same samples compounded with the poor fish hygienic production and raw fish consumption habit of the community suggest the potential risk of dissemination of the organisms along the supply chain and the transmission pathways from contaminated water-to-fish-to-humans. Further robust molecular study is required to establish the epidemiology of sorbitol non-fermenting *E. coli* strains along fish supply chain and characterize the new strains with unknown O-antigen to guide public health intervention programs to ensure fish safety in Ethiopia in general and in central Oromia in particular.

### Supplementary Information

Supplementary Information.

## Data Availability

The DNA Sequence datasets generated during the current study are available in the National Library of Medicine repository, with accession numbers: SUB13951691, SUB13951614, SUB13951611, SUB13951598, SUB13951583, SUB13951579, SUB13951565, SUB13951559, SUB13951552, SUB13951544, SUB13951396, SUB13951541, SUB13951294, SUB13951282, SUB13951298, SUB13951274, SUB13951308, SUB13951320, SUB13951382, SUB13951387, SUB13951393, SUB13942239, SUB13951735, SUB13951753, SUB13951569, SUB13949288, SUB13949315, SUB13949345, and SUB13949358. All other data were incorporated to the manuscript.
