# Supplementary material for: Occurrence, molecular characterization, and antimicrobial susceptibility of sorbitol non-fermenting Escherichia coli in lake water, fish and humans in central Oromia, Ethiopia
Source: Sci Rep. 2024 May 30;14:12461. doi: 10.1038/s41598-024-61810-z (PMC11139919; doi:10.1038/s41598-024-61810-z)
Supplement: Supplementary file 1 — Supplementary Information. [file 41598_2024_61810_MOESM1_ESM.pdf]

Supplementary file, S1: Whole Genome Sequencing (WGS) Result Showing Serotypes, Common Virulence Factors and AMR Traits of SN-F *E. coli* Strains Retrieved from Water, Fish and Humans in Central Oromia, Ethiopia

Sample Taken	Source	*E. coli* Serotype Detected	Number of Isolates with the Same Antigen	Virulence Traits	Locus	Antimicrobial Resistance Traits	Locus	AMR Identity Scores (%)
Water	Lake Babogaya	O116: H49	1	Adherence<br>Protease<br>Regulation<br>Toxin	gad, IpfA<br>k88ab<br>terC<br>hlyE	No Results	No Results	
	Lake Hora-Arsedi	O20: H8	1	Toxin<br>Protease<br>Adherence<br>Regulation<br>Complement<br>Protease<br>Survival<br>Invasion	hlyE<br>gad<br>lpfA<br>terC<br>traT<br>iss<br>ompT	No Results	No Results	
		O174: H43	1	Toxin<br>Protease<br>Adherence<br>Regulation<br>Survival<br>Invasion	hlyE<br>gad<br>lpfA<br>terC<br>iss<br>ompT, cib	Doxycycline<br>Trimethoprim<br>Sulfamethoxazole<br>Tetracycline	tet(A)<br>dfrA1<br>sul1<br>tet(A)	100<br>99.79<br>100<br>100
		O8: H10	1	Toxin<br>Protease<br>Adherence<br>Regulation	hlyE<br>gad<br>lpfA<br>terC	No Results	No Results	

	Lake Koftu	O116: H49	1	Toxin<br>Protease<br>Adherence<br>Regulation	hlyE<br>gad<br>k88ab, lpfA<br>terC	No Results	No Results	
	Lake Koka	O17/O44:<br>H18	1	Toxin<br>Protease<br>Adherence<br>Regulation<br>Survival<br>Invasion<br>Iron uptake<br>Complement<br>protease<br>Secretion system<br>[not specified]	cma, hlyE,<br>hlyF<br>gad, vat<br>lpfA, air<br>terC, eilA<br>iss<br>cia<br>chuA, sitA,<br>iroN<br>traT<br>kpsE,<br>kpsMll_k52<br>etsC	No Results	No Results	
	Lake Dambel	O17/O44:<br>H18	1	Toxin<br>Invasion<br>Protease<br>Adherence<br>Secretion system<br>Regulation<br>Iron uptake<br>Survival<br>Complement<br>Protease<br>[not specified]	cma<br>cia<br>vat, gad<br>air<br>kpsmll-k52<br>terC, eilA<br>sitA, chuA<br>iss<br>traT<br>etsC	No Results	No Results	

Fish skin swab	Lake Koka	O176:H11	1	Toxin<br>Adherence<br>Protease<br>Regulation<br>Iron uptake<br>Survival<br>Complement protease<br>Invasion	hlyE, hlyF,<br>cma, cvaC<br>lpfA<br>gad<br>terC<br>sitA, iroN<br>iss<br>traT<br>ompT	ampicillin<br>amoxicillin<br>Doxycycline<br>piperacillin<br>ticarcillin<br>cephalothin<br>Tetracycline	blaTEM-1B<br>blaTEM-1B<br>tet(A)<br>blaTEM-1B<br>blaTEM-1B<br>blaTEM-1B<br>tet(A)	100<br>100<br>100<br>100<br>100<br>100<br>100<br>100<br>100
Fish Meat	Lake Koftu	O18ac: H21	1	Toxin<br>Protease<br>Adherence<br>Regulation<br>Survival<br>Invasion	hlyE<br>gad<br>lpfA<br>terC<br>iss<br>cia	ampicillin<br>amoxicillin<br>cephalothin<br>Doxycycline<br>piperacillin<br>ticarcillin<br>Tetracycline<br>Minocycline	blaTEM-1B<br>blaTEM-1B<br>blaTEM-1B<br>tet(A)<br>blaTEM-1B<br>blaTEM-1B<br>tet(B)<br>cat (B)	100<br>100<br>100<br>100<br>100<br>100<br>100<br>100
	Lake Koka	O176: H11	2	Toxin<br>Protease<br>Regulation<br>Survival	hlyE<br>gad<br>terC<br>iss	ampicillin<br>amoxicillin<br>cephalothin<br>Doxycycline<br>piperacillin<br>ticarcillin<br>Tetracycline	blaTEM-1B<br>blaTEM-1B<br>blaTEM-1B<br>tet(A)<br>blaTEM-1B<br>blaTEM-1B<br>tet(B)	100<br>100<br>100<br>100<br>100<br>100<br>100
	Lake Koka	O155: H21	1	Toxin<br>Protease<br>Adherence<br>Regulation	hlyE<br>gad<br>lpfA<br>terC	No Results	No Results	
		O10: H5	1	Toxin	hlyE	Doxycycline	tet(A)	100

				Protease<br>Regulation	gad<br>terC	Tetracycline<br>Ciprofloxacin (mutational)<br>Nalidixic acid (mutational)	tet(A)<br>parC<br>parC	100<br>56 (position)<br>56 (position)
Fish feces	Lake Koka	O-unknown: H28	1	Toxin<br>Invasion<br>Protease<br>Regulation<br>Iron uptake<br>Survival	hlyE<br>ompT<br>gad<br>eilA, terC<br>chuA<br>iss	No Results	No Results	
Human stool	Koka Health Center	O168: H2	2	Toxin<br>Protease<br>Adherence<br>Regulation<br>Complement protease	hlyE<br>gad<br>lpfA<br>terC<br>traT	Fosfomycin	fosA7	94.09
		O8: H25	1	Toxin<br>Protease<br>Adherence<br>Regulation<br>Complement protease<br>Iron uptake<br>Invasion<br>Survival<br>[Not specified]	hlyE, hlyF, cvaC, mchF<br>gad<br>lpfA, irp2<br>terC<br>traT<br>fyuA, sitA, iroN, iutA<br>ompT, cia<br>iss<br>etsC	ampicillin<br>amoxicillin<br>cephalothin<br>Doxycycline<br>piperacillin<br>Trimethoprim<br>Sulfamethoxazole<br>Ticarcillin<br>Tetracycline<br>Nalidixic acid (mutational)	blaTEM-1B<br>blaTEM-1B<br>blaTEM-1B<br>tet(A)<br>blaTEM-1B<br>dfrA5<br>sul1<br>blaTEM-1B<br>tet(A)<br>gyrA	100<br>100<br>100<br>100<br>100<br>100<br>100<br>100<br>100<br>86

						Ciprofloxacin (mutational)	gyrA	86
	Bishoftu Hospital	O155: H10	1	Toxin<br>Adherence<br>Protease<br>Regulation<br>Iron uptake<br>Survival<br>Complement<br>protease<br>Invasion	hlyE, hlyF,<br>cma, cvaC<br>lpfA<br>gad<br>terC<br>sitA, iroN<br>iss<br>traT<br>ompT	ampicillin<br>amoxicillin<br>Doxycycline<br>piperacillin<br>Trimethoprim<br>Sulfamethoxazole<br>ticarcillin<br>cephalothin<br>Tetracycline<br>Ciprofloxacin<br>Chloramphenicol	blaTEM-1B<br>blaTEM-1B<br>tet(A)<br>blaTEM-1B<br>dfrA12<br>sul3<br>blaTEM-1B<br>blaTEM-1B<br>tet(A)<br>qnrS11<br>cmlA1	100<br>100<br>100<br>100<br>100<br>99.87<br>100<br>100<br>100<br>100<br>99.84
		O-unknown: H40	1	Toxin<br>Adherence<br>Protease<br>Regulation<br>Iron uptake<br>Survival	hlyE<br>irp2<br>gad<br>terC<br>fyuA<br>iss	ampicillin<br>amoxicillin<br>cephalothin<br>Doxycycline<br>piperacillin<br>Trimethoprim<br>Sulfamethoxazole<br>ticarcillin<br>Tetracycline<br>Nalidixic acid<br>Ciprofloxacin<br>Aztreonam<br>Cefepime<br>Ceftaxime<br>Ceftazidime<br>Ceftriaxone	blaTEM-1B<br>blaTEM-1B<br>blaTEM-1B<br>tet(A)<br>blaTEM-1B<br>dfrA40<br>sul2<br>blaTEM-1B<br>tet(A)<br>gyrA<br>qnrS1<br>blaCTX-M15<br>blaCTX-M15<br>blaCTX-M15<br>blaCTX-M15<br>blaCTX-M15	100<br>100<br>100<br>100<br>100<br>100<br>100<br>100<br>100<br>100<br>100<br>100<br>100<br>100<br>100<br>100

							(ESBL)	100<br>100<br>100<br>100<br>100<br>100
	Batu Health Center	O155: H10	4	Toxin<br>Adherence<br>Protease<br>Regulation<br>Iron uptake<br>Survival<br>Complement<br>protease<br>Invasion	hlyE, hlyF,<br>cma, cvaC<br>lpfA<br>gad<br>terC<br>sitA, iroN<br>iss<br>traT<br>ompT	ampicillin<br>amoxicillin<br>Doxycycline<br>piperacillin<br>Trimethoprim<br>Sulfamethoxazole<br>ticarcillin<br>cephalothin<br>Tetracycline<br>Ciprofloxacin<br>Chloramphenicol	blaTEM-1B<br>blaTEM-1B<br>tet(A)<br>blaTEM-1B<br>dfrA12<br>sul3<br>blaTEM-1B<br>blaTEM-1B<br>tet(A)<br>qnrS11<br>cmlA1	100<br>100<br>100<br>100<br>100<br>99.87<br>100<br>100<br>100<br>100<br>99.84

NB: No Result means no virulence traits /antimicrobial resistance determinant genes were detected.
